# Deficits in emotion recognition and processing in children with high callous-unemotional traits: the role of the *MAOA* gene

**DOI:** 10.1007/s00787-024-02397-8

**Published:** 2024-03-20

**Authors:** Pietro Muratori, Sara Palumbo, Stefano Vellucci, Veronica Mariotti, Lucia Billeci, Valentina Levantini, Emanuela Inguaggiato, Gabriele Masi, Annarita Milone, Silvia Pellegrini

**Affiliations:** 1 Scientific Institute of Child Neurology and Psychiatry, IRCCS Fondazione Stella Maris, Calambrone, Pisa, Italy; 2 Department of Clinical and Experimental Medicine, University of Pisa, Pisa, Italy; 3 Institute of Clinical Physiology, National Research Council of Italy (CNR), Pisa, Italy

**Keywords:** Callous-unemotional traits, Genetics, Eye-tracking, MAOA, Conduct problems

## Abstract

Children with high Callous-Unemotional (CU) traits show deficits in recognizing and processing facial expressions. Alterations in emotion recognition have been linked to a higher synaptic concentration of monoaminergic neurotransmitters. The current study investigated the relationship between the *MAOA*-Low-activity alleles and the ability to recognize and process facial expressions in 97 male children (8–12 years old) diagnosed with disruptive behavior disorder. Participants completed a computerized emotion-recognition task while an eye-tracking system recorded the number (Fixation Count, FC) and length (Fixation Duration, FD) of fixations to the eye region of the emotional stimuli. Children with high CU traits exhibited lower scores in recognition of sadness and anger, and lower FC and FD for sadness and fear than children with low CU traits. Children carrying the *MAOA*-Low-activity alleles displayed lower FD for sadness, and FD and FC for fear than those carrying the *MAOA*-High-activity alleles. These genetic effects appeared even stronger in children with CU traits. Moderation analysis revealed that CU traits were associated with lower FC and FD for fear, and lower FD for sadness, probably due to the *MAOA*-Low-activity alleles. Our findings, although to be replicated, suggest *MAOA*-Low-activity alleles as potential genetic biomarkers to identify CU children in need of training focused on emotion processing.

**Supplementary Information:**

The online version contains supplementary material available at 10.1007/s00787-024-02397-8.

## Introduction

Callous-unemotional (CU) traits involve a lack of guilt and remorse, a lack of concern for others’ feelings, shallow or superficial emotions, and indifference about performance on important tasks [1]. CU traits have significant theoretical and clinical implications as they differentiate youths with severe disruptive and aggressive behavior [2], whose behavioral problems emerge early, are persistent, and are resistant to treatment [3].

Youths with high CU traits show difficulty in recognizing sad and fearful faces [4–7], and this impairment might extend to other types of emotions [8]. In a groundbreaking study [9], CU traits in children and adolescents were associated with reduced attention to the eyes of fearful faces. Later studies reported impaired gaze patterns in children and adolescents with CU traits, showing reduced attention to the eyes of sad, fearful, and disgusted faces [5, 6, 10–13]. A recent study on twins showed that the relationship between high CU traits and deficits in recognizing aversive facial expressions in children, adolescents, and emerging adults is modulated by genetics [12]. The *MAOA* gene, encoding the monoamine oxidase enzyme, is a good candidate for studying the relationship between inter-individual variability in monoaminergic neurotransmission and emotion processing. MAOA catabolizes the monoamine neurotransmitters serotonin, noradrenaline, and dopamine, which play a significant role in mediating emotions [13]. An elevated synaptic concentration of neuroactive amines may alter emotion recognition by increasing the activation of the limbic areas and decreasing the prefrontal brain reaction during the passive observation of facial emotions [14, 15]. The promoter of the *MAOA* gene contains a Variable Number of Tandem Repeat (VNTR), with a 30 bp unit repeated 2, 3, 3.5, 4, or 5 times [16, 17]. The 2, 3, and 5 repeat alleles, known as Low-activity alleles, decrease the *MAOA* expression by 30%. These alleles have been extensively linked to increased impulsivity, novelty and sensation seeking, externalizing problems, antisocial, aggressive, and criminal behavior [18–20], and evidence of their association with high psychopathic traits has been reported both in youths and adults [21, 22].

Here, we investigated whether the *MAOA*-uVNTR alleles may be associated with deficits in emotion processing in youths with CU traits by studying their ability to recognize emotions and gaze patterns while observing facial images expressing different emotions.

## Methods

### Participants

Participants were 103 children (age: mean 9.21 ± 1.54 years, range 8–12 years; IQ-WISC IV [23]: mean 100.22 ± 8.46, range 80–130) with behavioral problems referred to a specialized service from 2019 to 2021. All participants received a diagnosis of Disruptive Behavior Disorder (DBD): 28 (27%) had a primary diagnosis of Conduct Disorder (CD) and 75 (73%) of Oppositional Defiant Disorder (ODD). Additionally, 70 of these youths (68%) were also diagnosed with Attention-Deficit Hyperactivity Disorder (ADHD). The inclusion criteria were as follows: a primary diagnosis of CD and/or ODD; Intelligence Quotient (IQ) ≥ 80; no ongoing medication treatment at the time of recruitment. Comorbidity with Autism Spectrum Disorder was an exclusion criterion. The study conformed to the principles of the Declaration of Helsinki and was approved by the Regional Ethical Committee (Meyer Hospital, Florence) (N. 64/2019).

### Measures

#### Categorical diagnosis

Children’s diagnosis was determined using the Kiddie Schedule for Affective Disorders and Schizophrenia for School-Age Children-Present and Lifetime Version (K-SADS-PL) [23]. Trained clinicians conducted the K-SADS-PL interviews. Both parents and children completed the K-SADS-PL interview independently. The rate of child-parent K-SADS-PL diagnosis agreement was 0.84 (k Cohen).

#### Intellectual functioning

Children’s cognitive abilities were assessed using the Wechsler Intelligence Scales for Children–4th Edition (WISC IV) [24], and an Intelligence Quotient (IQ) score was calculated for each child.

#### Callous-unemotional traits

CU traits were assessed by the Italian version of the Antisocial Process Screening Device (APSD) [25] parent-report. An APSD-CU cut-off score of six was applied to separate youths with high vs. low CU traits, according to the APSD manual. Thirty-seven (36%) patients scored six or higher on the APSD-CU questionnaire. The Cronbach’s alfa of the CU scale in the current sample was 0.78.

#### Emotion recognition and gaze pattern

The gaze pattern was recorded using the SMI RED 500 binocular eye-tracker (SensoMotoric Instruments; Teltow, Germany). Participants completed a computerized emotion recognition task with the eye-tracker in front of them, below a 22-inch flat-screen monitor, at about 65 cm. Children were presented with images from the NimStim Set of Facial Expressions [26], depicting happy, fearful, angry, disgusted, and sad facial expressions. An attention-getter was displayed before each trial to capture children’s attention.

Participants were asked to label the facial emotion displayed on the screen (Emotion Recognition, ER). Regarding gaze pattern, the outcome measures were the number of fixations (Fixation Count; FC) and the average length of fixation (Fixation Duration; FD) to the eye region, selected as the area of interest [6]. A fixation threshold of 100 ms was applied to the raw data to avoid unconscious looking. To adjust for individual differences due to blinking or momentary distraction from the screen, the FC and FD of the eye region were calculated as a percentage of the overall FC or FD of the whole face, respectively.

*Genotyping*. Each participant provided a saliva sample by an Oragene collection tube (DNA Genotek Inc., Ottawa, Ontario, Canada). DNA was extracted from saliva by the prepITL2P kit (DNA Genotek Inc.) and stored at − 20 °C. The *MAOA*-uVNTR sequence was amplified by Polymerase Chain Reaction (PCR protocol: 95 °C/15 min, 94 °C/30 s-62 °C/30 s-72 °C/60 s for 35 cycles, 72 °C/10 min) with the following primers: 5′-ACAGCCTGACCGTGGAGAAG-3′ and 5′-GAACGGACGCTCCATTCGGA-3′, and genotyped by comparison, on a 2% agarose gel, with the GeneRuler DNA ladder (Thermofisher Scientific, Waltham, MA, USA) (error rate: 0%; call rate: 100%). For the association analysis, two *MAOA*-uVNTR allele groupings were created to compare *MAOA-*Low-activity allele carriers (3r, *N* = 29, 27.6%) with *MAOA-*High-activity allele carriers (3.5r and 4r, *N* = 74, 70.1%) [16].

### Statistical analyses

Statistical analysis was performed using the SPSS 27 software package (IBM Corporation, Armonk, NY, USA).

#### Outliers

Multivariate outliers were detected by calculating the Mahalanobis distance [27]. Six subjects showed outliers for all the analyzed dependent variables (ER, FC, and FD) and were eliminated from the final sample (97 children).

#### Search for confounding factors

The Spearman’s rank correlation test was used to investigate whether age and IQ significantly influenced ER, FC, and FD. The Mann–Whitney U test was used to examine whether youths with APSD-CU scores ≥ 6 had significantly different ages and IQs from those with APSD-CU scores < 6. The Mann–Whitney U test was also used to examine whether ADHD influenced ER, FC, and FD. A Chi-square (Pearson) test was applied to investigate possible differences in the frequency of ADHD diagnoses and the *MAOA-*uVNTR alleles between youths with APSD-CU scores ≥ 6 and < 6.

*Association analysis:* To investigate whether the *MAOA*-uVNTR alleles predict emotion recognition deficits typical of children with CU traits, the nominal and interactive associations among the *MAOA*-uVNTR alleles, APSD-CU cut-off, and each dependent variable were investigated by the Multivariate Analysis of Variance (MANOVA). Partial eta squared values were reported, expressing the proportion of total variability attributable to each factor (i.e., *MAOA*-uVNTR alleles, APSD-CU cut-off, or their interaction). The Spearman’s rank correlation test explored collinearity among dependent variables. Deviation from a normal distribution was assessed by the Shapiro–Wilk test. The presence of heteroskedasticity was assessed using the SPSS HeteroskedasticityV3 macro [28]. The equality of covariance matrices of the dependent variables across groups was assessed using Box’s test, while Levene’s test assessed the equality error variance. Wild bootstrapping inference was applied to control for normality deviations and heteroskedasticity based on 5000 wild bootstrap samples with Bias-corrected and accelerated (Nca) and simple resampling method.

The observed power (1-β) of the effect of each factor (i.e., *MAOA*-uVNTR allele, APSD-CU cut-off, or their interaction) was calculated at the appropriate alpha level:

- αlevel = 0.05/[15 dependent variables (i.e., 5ER + 5FC + 5FD) × 2 independent variables (i.e., *MAOA*-uVNTR allele groupings and APSD-CU cut-off)] = 0.0017 for the nominal influence of the *MAOA*-uVNTR alleles or APSD-CU cut-off.
- αlevel = 0.05/[15 dependent variables (i.e., 5ER + 5FC + 5FD) = 0.003 for the nominal influence of the *MAOA*-uVNTR alleles by APSD-CU cut-off interaction.

To correct the post-hoc analysis of the *MAOA*-uVNTR alleles by APSD-CU cut-off interaction, the level of significance was set according to the Bonferroni method, considering the number of simultaneously tested hypotheses to limit the type I error:

αlevel = 0.05/[15 dependent variables (i.e., 5ER + 5FC + 5FD) × 6 *MAOA*-uVNTR allele groupings by APSD-CU cut-off comparisons (i.e., *MAOA*-Low/APSD-CU < 6 vs. *MAOA*-Low/APSD-CU ≥ 6 + *MAOA*-Low/APSD-CU < 6 vs. *MAOA*-High/APSD-CU ≥ 6 + *MAOA*-Low/APSD-CU < 6 vs. *MAOA*-High/APSD-CU < 6 + *MAOA*-High/APSD-CU < 6 vs. *MAOA*-High/APSD-CU ≥ 6 + *MAOA*-High/APSD-CU < 6 vs. *MAOA*-Low/APSD-CU ≥ 6 + *MAOA*-High/APSD-CU ≥ 6 + *MAOA*-Low/APSD-CU ≥ 6) = 0.0006. For each comparison, the effect size (Cohen’s coefficient, “d”) and the power (1-β) were calculated by a post hoc power analysis for a two-group independent sample t-test in G*power 3.1.9.2 software [29].

To address whether the *MAOA*-uVNTR alleles moderated the path from emotion recognition deficits to CU traits, we performed a moderation analysis using SPSS Process version 4.2 beta macro (http://www.afhayes.com/). We computed logistic regression analyses into a simple moderation model (Model 1), with APSD-CU cut-off as the dependent variable, ER, FC, or FD as independent variables, and the *MAOA*-uVNTR alleles as the moderators. Cribari-Neto correction and bootstrap inference (5000 resamplings) were applied to control for deviations from the normal distribution.

## Results

### Descriptive analyses and search for confounding factors

The mean and standard deviation of each variable, divided by APSD-CU cut-off or *MAOA*-uVNTR alleles, are reported in Supplementary Table 1, while descriptive data for each combination of APSD-CU cut-off and *MAOA*-uVNTR alleles in Supplementary Table 2.

Dependent variables were not significantly influenced by age, IQ, and ADHD diagnosis (Supplementary Table 3 and 4). Age, IQ, and ADHD diagnosis were not significantly associated with the APSD-CU cut-off (Supplementary Table 5 and 6).

#### *MAOA*-uVNTR alleles by APSD-CU cut-off interactions

The frequency of the *MAOA*-uVNTR alleles was not significantly different between youths with APSD-CU scores ≥ 6 and < 6 (*MAOA*-Low-activity alleles and APSD-CU scores ≥ 6: *N* = 13, *MAOA*-High-activity alleles and APSD-CU scores ≥ 6: *N* = 20, *MAOA*-Low-activity alleles and APSD-CU scores < 6: *N* = 14, *MAOA*-High-activity alleles and APSD-CU scores < 6: *N* = 50; OR = 2.32, Person’s Chi-square: 3.33, *p* = 0.07).

### Associations with emotion recognition (ER)

**Fig. 1:**
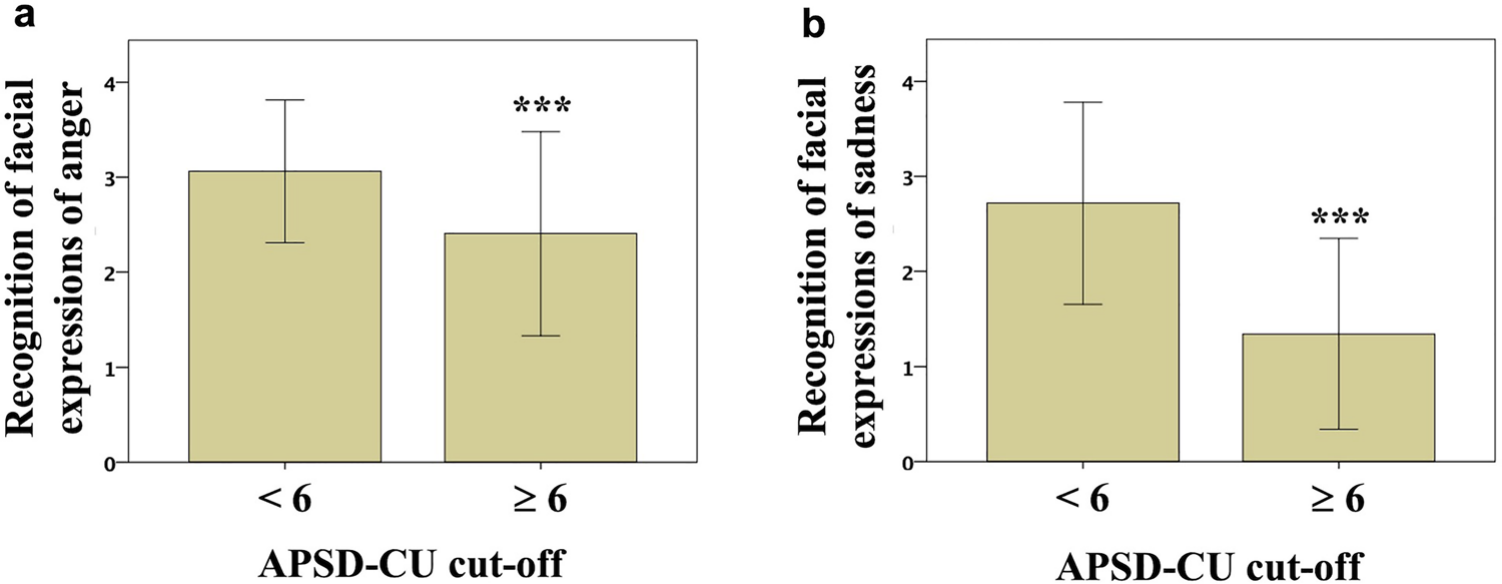
Direct association between APSD-CU cut-off and Emotion Recognition. Recognition of facial expressions of **a** anger and **b** sadness. Data are means  ±  1SD

- APSD-CU cut-off
  APSD-CU cut-off was significantly associated with ER_Anger (F1,96 = 14.105, *p* = 3.03 × 10^−4^; ηpartial^2^ = 0.133, 1-β = 0.697; Fig. 1a) and ER_Sadness scores (F1,96 = 38.961, *p* = 1.31 × 10^–8^; ηpartial^2^ = 0.298, 1-β = 0.998; Fig. 1b). In detail, ER_Anger and ER_Sadness scores were lower in youths with APSD-CU scores ≥ 6 (ER_Anger mean score: 2.41 ± 1.07; ER_Sadness mean score: 1.34 ± 1.00; *N* = 32) than in those with scores < 6 (ER_Anger mean score: 3.06 ± 0.75; ER_Sadness mean score: 2.72 ± 1.06; *N* = 64).
  The association between APSD-CU cut-off and ER_Happiness scores was significant (*p* = 2.34 × 10^–3^), but the statistical power was low (1-β = 0.463) (Table 1). ER_Fear and ER_Disgust scores were not significantly different between youths with APSD-CU scores ≥ 6 and < 6 (Table 1).

**Table 1:** MANOVA results testing the nominal and the interactive association between APSD-CU cut-off and *MAOA-*uVNTR alleles and Emotion Recognition (ER), Fixation Count (FC), and Fixation Duration (FD).

		APSD-CU cut-off			*MAOA-*uVNTR alleles			APSD-CU cut-off by *MAOA-*uVNTR alleles interaction		
		F	df	*p*	F	df	*p*	F	df	*p*
Emotion Recognition (ER)	ER_Anger	14.105	1.96	3.03 × 10^–4*^	1.797	1.96	0.183	3.403	1.96	0.068
	ER_Sandess	38.961	1.96	1.31 × 10^–8*^	0.116	1.96	0.734	2.225	1.96	0.139
	ER_Fear	0.000	1.96	0.989	0.618	1.96	0.434	2.675	1.96	0.105
	ER_Disgust	0.955	1.96	0.331	0.965	1.96	0.965	0.493	1.96	0.484
	ER_Happiness	9.797	1.96	2.34 × 10^–3^	2.686	1.96	0.105	5.128	1.96	0.026
Fixation Count (FC)	FC_Anger	7.219	1.95	0.009	2.407	1.95	0.124	6.105	1.95	0.015
	FC_Sandess	19.277	1.95	3.00 × 10^–5*^	10.460	1.95	0.002	11.211	1.95	0.001
	FC_Fear	18.843	1.95	3.70 × 10^–5*^	18.750	1.95	3.80 × 10^–5*^	18.751	1.95	3.80 × 10^–5*^
	FC_Disgust	6.936	1.95	0.010	0.797	1.95	0.374	1.690	1.95	0.197
	FC_Happiness	0.047	1.95	0.829	3.889	1.95	0.052	2.403	1.95	0.125
Fixation Duration (FD)	FD_Anger	9.387	1	0.004	2.212	1.95	0.140	6.474	1.95	0.013
	FD_Sandess	26.801	1	1.00 × 10^–6*^	14.778	1.95	2.24 × 10^–4*^	11.586	1.95	0.001*
	FD_Fear	18.243	1	4.80 × 10^–5*^	20.468	1.95	1.80 × 10^–5*^	14.347	1.95	2.72 × 10^–4*^
	FD_Disgust	7.836	1	0.006	0.408	1.95	0.525	1.985	1.95	0.162
	FD_Happiness	0.142	1	0.707	4.361	1.95	0.040	2.476	1.95	0.119

*p values below the alpha level of significance

b) *MAOA-*uVNTR alleles
  ER scores were not significantly different between carriers of the *MAOA*-Low-activity alleles and carriers of the *MAOA*-High-activity alleles (Table 1).

c) APSD-CU cut-off by *MAOA*-uVNTR alleles interaction
  The APSD-CU cut-off by *MAOA*-uVNTR alleles interaction did not significantly influence ER scores with the only exception of the ER_Happiness (*p* = 0.026), but the statistical power was low (1-β = 0.224) (Table 1).

### Associations with fixation counts (FC)

**Fig. 2:**
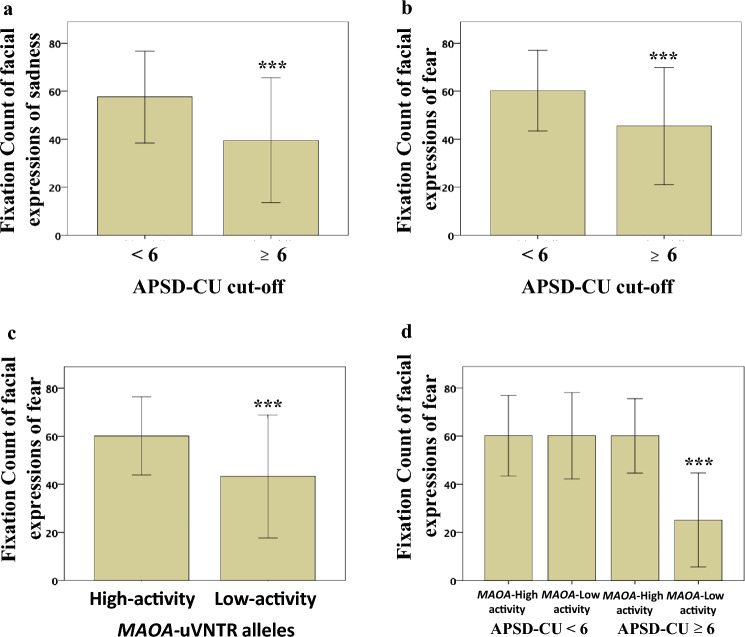
Nominal and interactive influence of APSD-CU cut-off and *MAOA-*uVNTR alleles on the Fixation Count of facial expressions. **a** Fixation Count of facial expressions of sadness in youths with APSD-CU scores < 6 and youths with APSD-CU scores ≥ 6. Fixation Count of facial expressions of fear in **b** youths with APSD-CU scores < 6 and youths with APSD-CU scores ≥ 6, **c** carriers of the *MAOA*-Low-activity alleles and carriers of the *MAOA*-High-activity alleles, and **d** youths with APSD-CU scores < 6 or APSD-CU scores ≥ 6 divided into carriers of the *MAOA*-Low-activity alleles or the *MAOA*-High-activity alleles. Data are means  ±  1SD

- APSD-CU cut-off
  APSD-CU cut-off was significantly associated with FC_Sadness (F1,95 = 19.277, *p* = 3.00 × 10^–5^; ηpartial^2^ = 0.175, 1-β = 0.870; Fig. 2a) and FC_Fear scores (F1,95 = 18.843, *p* = 3.7 × 10^–5^; ηpartial^2^ = 0.172, 1-β = 0.860; Fig. 2b).
  In detail, FC_Sadness and FC_Fear scores were lower in youths with APSD-CU scores ≥ 6 (FC_Sadness mean score: 38.51 ± 26.03; FC_Fear mean score: 45.47 ± 24.42; *N* = 31) than in those with scores < 6 (FC_Sadness mean score: 57.58 ± 19.18; FC_Fear mean score: 60.18 ± 16.84; *N* = 64).
  The association between APSD-CU cut-off and FC_Disgust scores was significant (*p* = 0.010), but the statistical power was low (1-β = 0.111) (Table 1). The association between APSD-CU cut-off and FC_Anger scores did not survive wild bootstrapping (*p* = 0.079) (Table 1). The association with FC_Happiness scores was not statistically significant (Table 1).

b) *MAOA*-uVNTR alleles
  *MAOA*-uVNTR alleles significantly influenced FC_Fear scores (F1,95 = 18.750, *p* = 3.8 × 10^− 5^; ηpartial^2^ = 0.171, 1-β = 0.857; Fig. 2c). In detail, FC_Fear scores were lower in carriers of the *MAOA*-Low-activity alleles (FC_Fear mean score: 43.32 ± 25.62; *N* = 27) than in carriers of the *MAOA*-High-activity alleles (FC_Fear mean score: 60.17 ± 16.27; *N* = 68).
  The association between the *MAOA-*uVNTR alleles and FC_Sadness scores was significant (*p* = 0.001), but the statistical power was low (1-β = 0.501) (Table 1). The associations with FC_Anger, FC_Disgust, and FC_Happiness scores were not statistically significant (Table 1).

c) APSD-CU cut-off by *MAOA*-uVNTR alleles interaction
  The interaction between APSD-CU cut-off and *MAOA*-uVNTR alleles significantly influenced FC_Fear scores (F1,95 = 18.751, *p* = 3.8 × 10^–5^; ηpartial^2^ = 0.171, 1-β = 0.896; Fig. 2d).
  The post-hoc analysis showed that FC_Fear scores were lower in carriers of the *MAOA*- Low-activity alleles with APSD-CU scores ≥ 6 (25.16 ± 19.58; *N* = 13) as compared to a) carriers of the *MAOA*-Low-activity alleles with APSD-CU scores < 6 (60.18 ± 17.97; *N* = 14; *p* = 4.05 × 10^–7^, *p*Bonferroni-corrected = 3.65 × 10^–5^; *d*Cohen = 1.5, 1-β = 0.67), b) carriers of the *MAOA*- High-activity alleles with APSD-CU scores ≥ 6 (60.14 ± 15.45; *N* = 18; *p* = 1.15 × 10^–7^, *p*Bonferroni-corrected = 1.03 × 10^–5^; *d*Cohen = 2.00, 1-β = 0.96), and c) carriers of the *MAOA*-High- activity alleles with APSD-CU scores < 6 (60.18 ± 16.71; *N* = 50; *p* = 7.81 × 10^–9^, *p*Bonferroni- corrected = 7.11 × 10^–7^; *d*Cohen = 1.94, 1-β = 0.99).
  The associations with FC_Sadness (*p* = 0.001) and FC_Anger scores (*p* = 0.015) were significant, but the statistical power was low (1-β = 0.618 and 0.289, respectively) (Table 1). The associations with FC_Disgust and FC_Happiness scores were not statistically significant (Table 1).

### Associations with fixation duration (FD)

**Fig. 3:**
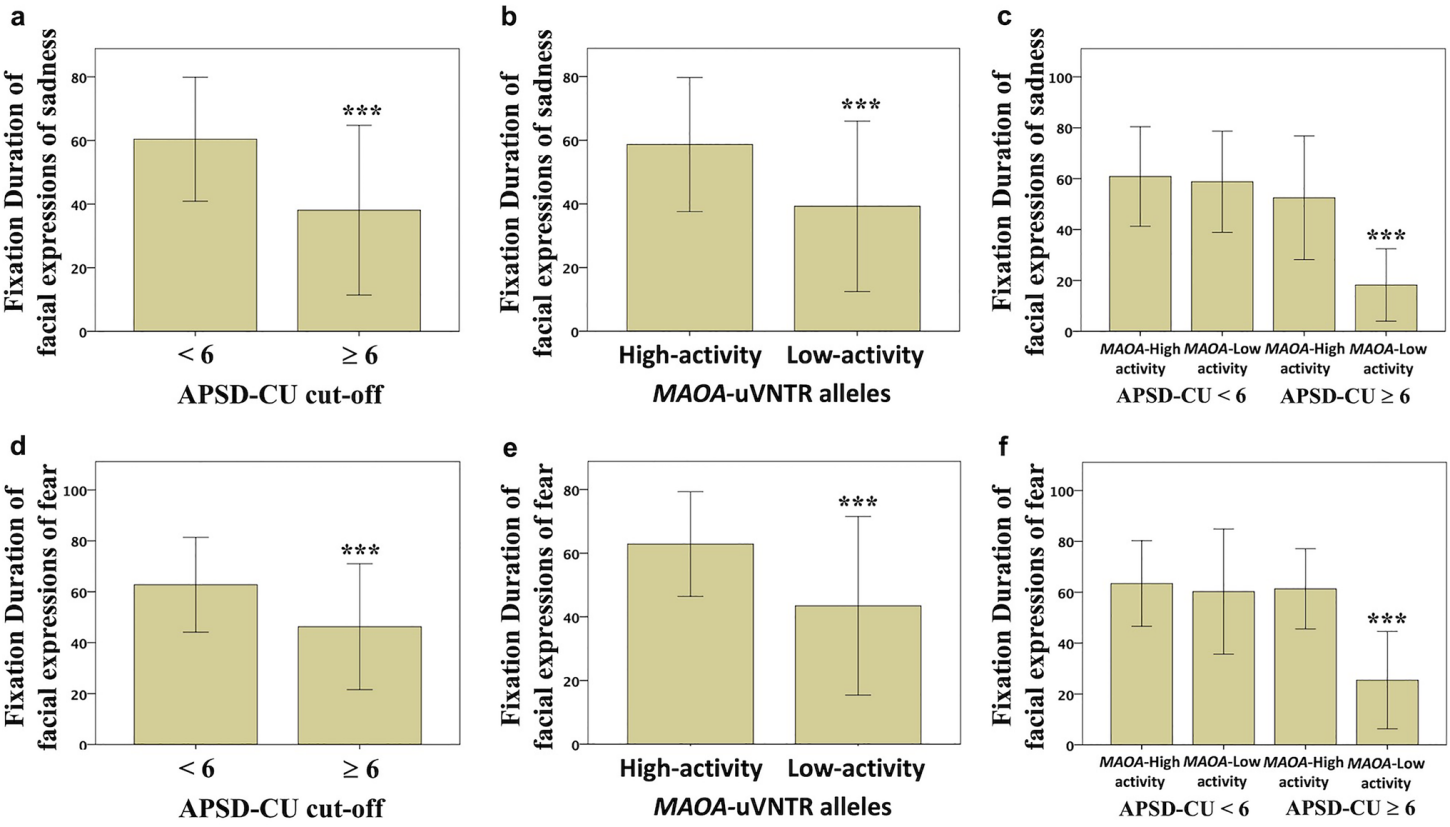
Nominal and interactive influence of APSD-CU cut-off and *MAOA-*uVNTR alleles on the Fixation Duration of facial expressions. Fixation Duration of facial expressions of sadness in **a** youths with APSD-CU scores < 6 and youths with APSD-CU scores ≥ 6, **b** carriers of the *MAOA*- Low-activity alleles and carriers of the *MAOA*-High-activity alleles, and **c** youths with APSD-CU scores < 6 or APSD-CU scores ≥ 6 divided into carriers of the *MAOA*-Low-activity alleles or the *MAOA*-High-activity alleles. Fixation Duration of facial expressions of fear in **d** youths with APSD-CU scores < 6 and youths with APSD-CU scores ≥ 6, **e** carriers of the *MAOA*-Low-activity alleles and carriers of the *MAOA*-High-activity alleles, and **f** youths with APSD-CU scores < 6 or APSD- CU scores ≥ 6 divided into carriers of the *MAOA*-Low-activity alleles or the *MAOA*-High-activity alleles. Data are means  ±  1SD

- APSD-CU cut-off
  APSD-CU cut-off was significantly associated with FD_Sadness (F1,95 = 26.801, *p* = 1 × 10^–6^; ηpartial^2^ = 0.228, 1-β = 0.971; Fig. 3a) and FD_Fear scores (F1,95 = 18.243, *p* = 4.8 × 10^–5^; ηpartial^2^ = 0.167, 1-β = 0.845; Fig. 3d).
  In detail, FD_Sadness and FD_Fear scores were lower in youths with APSD-CU scores ≥ 6 (FD_Sadness mean score: 38.11 ± 26.70; FD_Fear mean score: 46.28 ± 24.73; *N* = 31) than in those with scores < 6 (FD_Sadness mean score: 60.42 ± 24.50; FD_Fear mean score: 62.73 ± 18.62; *N* = 64).
  The association with FD_Anger scores was significant (*p* = 0.004; Table 1) but did not survive wild bootstrapping (*p* = 0.051). The association with FD_Disgust scores was significant (*p* = 0.006; Table 1), but the power was low (1-β = 0.339). The association with FD_Happiness scores was not statistically significant (Table 1).

b) *MAOA*-uVNTR alleles
  *MAOA*-uVNTR alleles significantly influenced FD_Sadness (F1,95 = 14.778, *p* = 2.24 × 10^-4^; ηpartial^2^ = 0.140, 1-β = 0.726; Fig. 3b) and FD_Fear scores (F1,95 = 20.468, *p* = 1.8 × 10^-5^; ηpartial^2^ = 0.184, 1-β = 0.897; Fig. 3e).
  In detail, FD_Sadness and FD_Fear scores were lower in carriers of the *MAOA*-Low- activity alleles (FD_Sadness mean score: 39.24 ± 26.80; FD_Fear mean score: 43.50 ± 28.03; N = 27) than in carriers of the *MAOA*-High-activity alleles (FD_Sadness mean score: 58.65 ± 21.07; FD_Fear mean score: 62.87 ± 16.46; N = 68).
  The association with FD_Happiness scores was significant (*p* = 0.04; Table 1), but the statistical power was low (1-β = 0.134). The associations with FD_Anger and FD_Disgust scores were not statistically significant (Table 1).

c) APSD-CU cut-off by *MAOA*-uVNTR alleles interaction
  The interaction between APSD-CU cut-off and *MAOA*-uVNTR allele significantly influenced FD_Sadness (F1,95 = 11.586, *p* = 1 × 10^–3^; ηpartial^2^ = 0.113, 1-β = 0.920; Fig. 3c) and FD_Fear scores (F1,95 = 14.347, *p* = 2.72 × 10^–4^; ηpartial^2^ = 0.136, 1-β = 0.963; Fig. 3f).
  The post-hoc analysis showed that FD_Sadness scores were lower in carriers of the *MAOA*-Low-activity alleles with APSD-CU scores ≥ 6 (18.20 ± 14.21; *N* = 13) than a) carriers of the *MAOA*-Low-activity alleles with APSD-CU scores < 6 (58.79 ± 19.89; *N* = 14; *p* = 9.07 × 10^–7^, *p*Bonferroni-corrected = 8.160 × 10^–5^; *d*Cohen = 2.35, 1-β = 0.99), b) carriers of the *MAOA*- High-activity alleles with APSD-CU scores ≥ 6 (52.48 ± 24.32; *N* = 18; *p* = 9.00 × 10^–6^, *p*Bonferroni-corrected = 8.100 × 10^–4^; *d*Cohen = 1.72, 1-β = 0.86), and c) carriers of the *MAOA*-High- activity alleles with APSD-CU scores < 6 (60.87 ± 19.57; *N* = 50; *p* = 8.28 × 10^–10^, *p*Bonferroni- corrected = 7.46 × 10^–8^; *d*Cohen = 2.50, 1-β = 0.99).
  As regards FD_Fear scores, the post-hoc analysis showed that it was lower in carriers of the *MAOA*-Low-activity alleles with APSD-CU scores ≥ 6 (25.42 ± 19.13; *N* = 13) as compared to (a) carriers of the *MAOA*-Low-activity alleles with APSD-CU scores < 6 (60.24 ± 24.59; *N* = 14; *p* = 3.00 × 10^-6^, *p*Bonferroni-corrected = 2.7 × 10^-4^; *d*Cohen = 1.58, 1-β = 0.67), (b) carriers of the *MAOA*-High-activity alleles with APSD-CU scores ≥ 6 (61.34 ± 15.76; *N* = 18; *p* = 5.21 × 10^-7^, *p*Bonferroni-corrected = 4.69 × 10^−5^; *d*Cohen = 2.05, 1-β = 0.97), and (c) carriers of the *MAOA*-High-activity alleles with APSD-CU scores < 6 (63.43 ± 16.82; *N* = 50; *p* = 1.82 × 10^-9^, *p*Bonferroni-corrected = 1.63 × 10^−7^; *d*Cohen = 2.11, 1-β = 0.99).
  The association with FD_Anger scores was significant (F1,95 = 6.474, *p* = 0.013; ηpartial^2^ = 0.066, 1-β = 0.711), but did not survive the Bonferroni correction (Table 2). The associations with FD_Disgust and FD_Happiness scores were not statistically significant (Table 1).

**Table 2:** Post-hoc results (*p* values) testing the interaction between APSD-CU cut-off and *MAOA-*uVNTR alleles on Emotion Recognition (ER), Fixation Count (FC), and Fixation Duration (FD)

		*MAOA-*Low-activity alleles APSD-CU < 6	*MAOA-*High-activity alleles APSD-CU ≥ 6	*MAOA-*Low-activity alleles APSD-CU ≥ 6
*MAOA-*High-activity alleles APSD-CU < 6	FC_Fear	0.778	0.754	7.81 × 10^–9^*
	FD_Anger	0.410	0.656	0.001
	FD_Sandess	0.731	0.130	8.28 × 10^–10^*
	FD_Fear	0.566	0.678	1.82 × 10^–9^*
*MAOA-*Low-activity alleles APSD-CU < 6	FC_Fear		0.994	4.05 × 10^–7^*
	FD_Anger		0.298	0.001
	FD_Sandess		0.379	9.07 × 10^–7^*
	FD_Fear		0.867	3.00 × 10^–6^*
*MAOA-*High-activity alleles APSD-CU ≥ 6	FC_Fear			1.15 × 10^–7^*
	FD_Anger			0.010
	FD_Sadness			9.00 × 10^–6^*
	FD_Fear			5.21 × 10^–7^*

*p values below the alpha level of significance

### Moderation analyses

The *MAOA*-uVNTR alleles significantly moderated the effect of FC_Fear (likelihood ratio test of highest order unconditional interaction: Wald Chi-square = 8.368, df = 1, *p* = 0.0138), FD_Fear (likelihood ratio test of highest order unconditional interaction: Wald Chi-square = 4.747, df = 1, *p* = 0.029), and FD_Sadness (likelihood ratio test of highest order unconditional interaction: Wald Chi-square = 9.319, df = 1, *p* = 0.0023) scores on the presence of CU traits. In detail, FC_Fear, FD_Fear, and FD_Sadness scores predicted an APSD-CU score ≥ 6 in the presence of the *MAOA-*Low-activity alleles (FC_FE: Z = − 2.8956, *p* = 0.004, bootstrap 95% CI − 3.659 to − 0.023; FD_Fear: Z = − 2.694, *p* = 0.007, bootstrap 95% CI − 2.723 to − 0.007;

FD_Sadness: Z = − 2.651, *p* = 0.008, bootstrap 95% CI − 6.528 to − 0.036), but not in the presence of the *MAOA-*High-activity alleles (FC_Fear: Z = 0.0096, *p* = 0.992; FD_Fear: Z = − 0.464, *p* = 0.6424; FD_Sadness: Z = − 1.239, *p* = 0.1502).

## Discussion

The scientific literature has consistently reported deficits in emotion recognition and processing among youths with DBD and high CU traits [5, 6, 10, 11, 30], highlighting the necessity to better understand these impairments and their underpinnings. The current study first explored the influence of CU traits on emotion recognition and processing in a sample of children with DBD diagnosis. Our data revealed that children with high CU traits were significantly less accurate in recognizing sad and angry facial expressions than those with low CU traits. Poorer happiness recognition was also observed, albeit with limited statistical power. Our findings corroborate previous results demonstrating how children with high CU traits are less accurate in recognizing emotions with a negative valence [5, 6, 9] but also face challenges in identifying positive emotions [8]. Furthermore, we found significantly lower fixation count and duration for sad and fearful expressions in children with high CU traits. These results indicate a strong association between CU traits and reduced attention to the eyes of fearful and sad expressions, in line with the existing scientific literature [5, 6, 9, 11, 31].

These difficulties might be related to some of the peculiar features of children with CU traits, including impairments in reward and punishment processing, aggressive behavior and poorer concern for others, and low prosociality [32–34]. For instance, poorer anger recognition might prevent children from properly responding to parenting strategies and common disciplinary behaviors used to correct aggressive behavior. As suggested by Dadds and Salmon [35], punishment insensitivity tends to gradually disrupt parenting strategies, leading to an escalation from mild to ineffective severe punishments, which contribute to higher rates of aggressive and antisocial behavior. Instead, difficulties in properly processing and recognizing sad and/or fearful expressions could contribute to the tendency of children with CU traits not to care for others nor act prosocially. Indeed, if one cannot decode and read other people’s emotional signals, they will not be able to empathetically respond to others’ needs [36–38].

The current study also investigated the potential role of the *MAOA*-uVNTR alleles in emotion recognition and processing (i.e., gaze pattern) deficits and further explored whether the *MAOA*-uVNTR alleles moderated the relationship between emotion recognition, gaze pattern, and CU traits in the same clinical sample of children with DBD.

Results showed that children carrying the *MAOA*-Low-activity alleles displayed lower attention to the eyes of sad and fearful faces than carriers of the *MAOA*-High-activity alleles. Specifically, the large effect sizes explained about 14% of the variance in fixation duration to sad expressions and 17% in both fixation count and duration to fearful ones.

The interaction between high APSD-CU scores and *MAOA*-Low-activity alleles accounted for additional 11%, 17%, and 14% increases in the explained variance of fixation duration to sad expressions, and fixation count and duration to fearful faces, respectively. These data were corroborated by moderation analyses, which revealed that CU traits were associated with lower attention to the eyes of sad and fearful expressions, probably due to the *MAOA*-Low-activity alleles.

Our study suggests that the *MAOA*-Low-activity alleles are especially associated with lower attention to the eyes of fearful expressions, and this evidence is further supported by the results of the interaction and moderation analyses. Based on the Violent Inhibition Mechanism (VIM) Model [39], individuals are equipped with a cognitive process for the control of conspecific aggression activated by distress signals (i.e., fearful and sad expressions) [39, 40]. Once aroused, the VIM leads to behavioral schemes that stop the perpetrators from attacking; this mechanism is thought to foster the development of moral emotions and, at the same time, inhibit aggressive and violent behavior, representing an important precursor of moral development. Biological factors, like the *MAOA*-Low-activity variants, might hinder children’s ability to pay attention to relevant emotional cues (e.g., fearful and sad eyes), ultimately preventing the VIM from being activated. If children cannot properly process others’ distress cues, the VIM will not unfold, and they will likely not retreat from the action that is causing harm to others. More importantly, early impairments in emotion processing might compromise the child’s moral development and lead to aggressive behavior, lack of empathy and remorse, poor prosociality, and a reduced interest in others’ feelings and well-being, which are frequently observed in children with CU traits.

Consistently with this hypothesis, growing findings point to a possible role of the *MAOA* alleles in emotion processing and recognition. MAOA is a key enzyme for the catabolism of monoaminergic neurotransmitters, including serotonin, noradrenaline, and dopamine. The *MAOA*-Low-activity alleles decrease the MAOA expression by 30%, thus increasing neurotransmitter concentration in the synaptic cleft [16, 17].

Pharmacological treatment with d,l-fenfluramine, which primarily increases the serotonin and, to a lesser extent, the dopamine release, reduces the ability to experience both positive and negative emotions in humans [41]. Additionally, electrophysiological studies demonstrated that the increasing of serotonin by the serotonin reuptake inhibitor citalopram alters the cortical processing of emotionally relevant stimuli, resulting in a response suppression to unpleasant visual images [42] and in reduced amygdala activation to fearful facial expressions [43]. In addition, the pharmacological enhancement of serotonergic and noradrenergic neurotransmission globally decreases brain activation in response to unpleasant images [44]. Concerning dopamine, evidence exists of its involvement in fear processing [45], though defining its role is complex. The inhibition of dopamine receptors with haloperidol, for example, has been shown to enhance the ability to recognize emotions in individuals with low basal dopamine levels and to reduce it in subjects with high basal dopamine levels [46].

Carriers of the *MAOA*-Low-activity alleles exhibit abnormalities in the connectivity between the cortex and amygdala [15]. Moreover, increased surface areas of the right basolateral nucleus of the amygdala and the right anterior cortical amygdaloid nucleus have been observed in antisocial individuals carrying these alleles [47]. Of note, these structural changes were also associated with high psychopathic traits [47]. The amygdala, together with the pulvinar and the insula, plays a significant role in the face recognition network and is specifically involved in processing facial emotion expressions [48, 49], particularly emphasizing fear [8].

We hypothesize that the higher brain extracellular monoamine concentration due to the *MAOA*-Low-activity alleles might reduce the capability of recognizing sad and fearful facial expressions by inducing structural changes in the amygdala and functional alterations in both the amygdala and cortex that may impact their connectivity. These changes might precede and underlie the development of CU traits, representing a risk for greater aggressive and violent behavior, poor empathetic concern, and low prosociality. Future longitudinal studies are warranted to corroborate this hypothesis.

## Conclusions

The results of the current study need to be interpreted considering some limitations, including the relatively small sample size and the cross-sectional design. Additionally, sample individual ancestry data, useful to accurately interpret genetic risk [50], were self-reported and not estimated through genome sequencing. Moreover, relevant contextual variables, which might also influence the link between emotion recognition impairments and CU traits [51], were unavailable. The growth environment, in interaction with genetics, is known to exert a relevant role in modulating behavior [52–54].

Finally, we employed a candidate gene approach—more prone to false positive results and less informative than genome-wide studies (GWAS) [55, 56]. However, solid evidence from scientific literature sustained the validity of the *MAOA*-uVNTR as a candidate gene variant able to affect emotional deficits [21, 22]. Moreover, the obtained *p*-values, corrected by the Bonferroni method, were as small as close to the threshold generally applied for genome-wide studies [57].

Despite its limitations, the current study may have relevant clinical implications. DBD is an umbrella concept encompassing a wide range of manifestations, and literature has pointed out the limits of the traditional disorder-centered and symptom-based classifications of mental disorders in unraveling the heterogeneity of DBDs and providing insights into the prevention and treatment of such disorders [48]. This calls for novel frameworks to study them, like the Research Domain Criteria (RDoC) initiative to investigate mental disorders in the context of the major domains of basic neurobehavioral functioning rather than within established diagnostic categories (https://www.nimh.nih.gov/research/research-funded-by-nimh/rdoc/about-rdoc) [58].

We explored the association between CU traits and impaired emotion recognition and processing, which represents a mechanism highly related to social processing according to the RDoC. Our results provided further evidence of severe impairment of this ability in children with DBDs and CU traits. Moreover, going beyond the existing literature, our findings, although preliminary and to be replicated, showed that the *MAOA-*Low-activity alleles are involved in the emotion recognition deficits associated with CU traits, suggesting them as potential genetic biomarkers useful to identify youths with DBDs at greater risk for such impairment. Children and adolescents with CU traits are typically, less responsive to traditional treatments. However, scientific evidence suggests that these children may benefit from interventions focused on emotion processing [59, 60], as supported by the recent development of interventions addressing the emotion processing deficits and the impaired sensitivity for emotional distress associated with CU traits [61, 62]. Our findings might help identify children who are more in need of training focused on emotion processing.

## Supplementary Information

Below is the link to the electronic supplementary material.

Supplementary file1 (DOCX 43 KB)** Supplementary Table 1.** Descriptive data of age, IQ, and the dependent variables (Emotion Recognition, Fixation Count, and Fixation Duration) divided by APSD-CU cut-off or *MAOA*-uVNTR allele groupings. **Supplementary Table 2.** Descriptive data of age, IQ, and the dependent variables (Emotion Recognition, Fixation Count, and Fixation Duration) divided by the combinations of APSD-CU cutoff and *MAOA*-uVNTR alleles. **Supplementary Table 3.** Search for confounding factors. Spearman’s rank correlations between the dependent variables (Emotion Recognition, Fixation Count, and Fixation Duration) and age and IQ. **Supplementary Table 4.** Search for confounding factors. Mann-Whitney U test between youths with and without ADHD for the dependent variables (Emotion Recognition, Fixation Count, and Fixation Duration). **Supplementary Table 5.** Search for confounding factors. Mann-Whitney U test for age and IQ between youths with APSD-CU scores ≥ 6 and youths with APSD-CU scores < 6. **Supplementary Table 6.** Search for confounding factors. Chi-square (Pearson) test to compare the frequency of ADHD diagnosis in youths with APSD-CU scores ≥ 6 with that in youths with APSD- CU scores < 6.

## Data Availability

The raw data utilized for this study cannot be openly shared to ensure the utmost protection of study participant privacy. However, special requests to access the data can be sent to the corresponding author, who will then seek permission. Participant consent to conduct further analyses on their data was obtained prior to data collection. Of note, shared data should not be disseminated, and their use should be restricted to the specific purpose for which access has been requested.
