# Supplementary material for: Deficits in emotion recognition and processing in children with high callous-unemotional traits: the role of the MAOA gene
Source: Eur Child Adolesc Psychiatry. 2024 Mar 20;33(10):3529–40. doi: 10.1007/s00787-024-02397-8 (PMC11564231; doi:10.1007/s00787-024-02397-8)
Supplement: Supplementary file 1 — Supplementary file1 (DOCX 43 KB) Supplementary Table 1. Descriptive data of age, IQ, and the dependent variables (Emotion Recognition, Fixation Count, and Fixation Duration) divided by APSD-CU cut-off or MAOA-uVNTR allele groupings. Supplementary Table 2. Descriptive data of age, IQ, and the dependent variables (Emotion Recognition, Fixation Count, and Fixation Duration) divided by the combinations of APSD-CU cutoff and MAOA-uVNTR alleles. Supplementary Table 3. Search for confounding factors. Spearman’s rank correlations between the dependent variables (Emotion Recognition, Fixation Count, and Fixation Duration) and age and IQ. Supplementary Table 4. Search for confounding factors. Mann-Whitney U test between youths with and without ADHD for the dependent variables (Emotion Recognition, Fixation Count, and Fixation Duration). Supplementary Table 5. Search for confounding factors. Mann-Whitney U test for age and IQ between youths with APSD-CU scores ≥ 6 and youths with APSD-CU scores < 6. Supplementary Table 6. Search for confounding factors. Chi-square (Pearson) test to compare the frequency of ADHD diagnosis in youths with APSD-CU scores ≥ 6 with that in youths with APSD- CU scores < 6. [file 787_2024_2397_MOESM1_ESM.docx]

**Journal:** [**European Child & Adolescent Psychiatry**](https://www.springer.com/journal/787)

**Title: Emotion Recognition and Processing Deficits in Children with High Callous-Unemotional Traits: The Role of the MAOA Gene**

^a^Pietro Muratori*,^b^Sara Palumbo*^, ^b^Stefano Vellucci, ^b^Veronica Mariotti, ^c^Lucia Billeci, ^a^Valentina Levantini, ^a^Emanuela Inguaggiato, ^a^Gabriele Masi, ^a^Annarita Milone, ^b^Silvia Pellegrini

^*^These authors equally contributed to the work and are listed in an alphabetical order

^Corresponding author: sara.palumbo@unipi.it

^a^IRCCS Fondazione Stella Maris, Scientific Institute of Child Neurology and Psychiatry,Calambrone, Pisa, Italy

^b^Department of Clinical and Experimental Medicine, University of Pisa, Italy

^c^Institute of Clinical Physiology, National Research Council of Italy (CNR), Pisa, Italy

|  |  | APSD-CU < 6 | | | APSD-CU ≥ 6 | | | *MAOA*-uVNTR High-activity alleles carriers | | | *MAOA*-uVNTR Low-activity alleles carriers | | |
| --- | --- | --- | --- | --- | --- | --- | --- | --- | --- | --- | --- | --- | --- |
|  |  | Mean | SD | N | Mean | SD | N | Mean | SD | N | Mean | SD | N |
|  | Age | 9.03 | 1.45 | 64 | 9.61 | 1.75 | 33 | 9.16 | 1.42 | 70 | 9.41 | 1.93 | 27 |
|  | IQ | 100.37 | 8.67 | 63 | 99.88 | 8.62 | 33 | 99.86 | 7.77 | 69 | 101.07 | 10.58 | 27 |
| Emotion Recognition  Score (ER) | ER_Anger | 3.06 | 0.75 | 64 | 2.41 | 1.07 | 32 | 2.93 | 0.80 | 70 | 2.62 | 1.16 | 26 |
|  | ER_Sandess | 2.72 | 1.06 | 64 | 1.34 | 1.00 | 32 | 2.29 | 1.24 | 70 | 2.19 | 1.20 | 26 |
|  | ER_Fear | 2.13 | 1.23 | 64 | 1.97 | 1.31 | 32 | 2.04 | 1.21 | 70 | 2.15 | 1.38 | 26 |
|  | ER_Disgust | 2.42 | 1.21 | 64 | 2.22 | 1.24 | 32 | 2.36 | 1.19 | 70 | 2.35 | 1.29 | 26 |
|  | ER_Happiness | 3.55 | 0.69 | 64 | 3.06 | 1.11 | 32 | 3.47 | 0.76 | 70 | 3.15 | 1.12 | 26 |
| Fixation Count  (FC) | FC_Anger | 57.37 | 18.24 | 64 | 46.60 | 23.79 | 31 | 55.17 | 20.44 | 68 | 48.17 | 20.57 | 27 |
|  | FC_Sandess | 57.58 | 19.18 | 64 | 38.51 | 26.03 | 31 | 56.15 | 21.62 | 68 | 40.41 | 23.38 | 27 |
|  | FC_Fear | 60.18 | 16.84 | 64 | 45.47 | 24.42 | 31 | 60.17 | 16.27 | 68 | 43.32 | 25.62 | 27 |
|  | FC_Disgust | 48.25 | 19.48 | 64 | 37.45 | 19.56 | 31 | 46.21 | 18.66 | 68 | 40.98 | 23.20 | 27 |
|  | FC_Happiness | 51.72 | 19.16 | 64 | 51.58 | 21.56 | 31 | 53.79 | 18.80 | 68 | 46.24 | 21.74 | 27 |
| Fixation Duration  (FD) (sec) | FD_Anger | 57.52 | 18.46 | 64 | 45.76 | 25.42 | 31 | 55.75 | 20.58 | 68 | 48.49 | 23.50 | 27 |
|  | FD_Sandess | 60.42 | 24.50 | 64 | 38.11 | 26.70 | 31 | 58.65 | 21.07 | 68 | 39.24 | 26.80 | 27 |
|  | FD_Fear | 62.73 | 18.62 | 64 | 46.28 | 24.73 | 31 | 62.87 | 16.46 | 68 | 43.50 | 28.03 | 27 |
|  | FD_Disgust | 49.48 | 20.40 | 64 | 3.02 | 19.28 | 31 | 46.94 | 18.49 | 68 | 42.73 | 25.50 | 27 |
|  | FD_Happiness | 52.78 | 20.70 | 64 | 51.67 | 21.87 | 31 | 54.88 | 19.35 | 68 | 46.22 | 23.89 | 27 |

**Supplementary Table 1.** Descriptive data of age, IQ, and the dependent variables (Emotion Recognition, Fixation Count, and Fixation Duration) divided by APSD-CU cut-off or *MAOA* uVNTR allele groupings. IQ= Intelligence Quotient.

|  |  | APSD-CU < 6 and  *MAOA*-uVNTR High-activity alleles carriers | | | APSD-CU < 6  and  *MAOA*-uVNTR Low-activity alleles carriers | | | APSD-CU ≥ 6 and  *MAOA*-uVNTR High-activity alleles carriers | | | APSD-CU ≥ 6 and  *MAOA*-uVNTR Low-activity alleles carriers | | |
| --- | --- | --- | --- | --- | --- | --- | --- | --- | --- | --- | --- | --- | --- |
|  |  | Mean | SD | N | Mean | SD | N | Mean | SD | N | Mean | SD | N |
|  | Age | 9.04 | 1.50 | 50 | 9.00 | 1.30 | 14 | 9.45 | 1.19 | 20 | 9.85 | 2.41 | 13 |
|  | IQ | 99.45 | 8.08 | 49 | 103.57 | 10.17 | 14 | 100.85 | 7.05 | 20 | 98.38 | 10.75 | 13 |
| Emotion Recognition  Score (ER) | ER_Anger | 3.04 | 0.78 | 50 | 3.14 | 0.77 | 14 | 2.65 | 0.88 | 20 | 2.00 | 1.28 | 12 |
|  | ER_Sandess | 2.62 | 1.11 | 50 | 3.07 | 0.83 | 14 | 1.45 | 1.19 | 20 | 1.17 | 0.58 | 12 |
|  | ER_Fear | 2.18 | 1.17 | 50 | 1.93 | 1.44 | 14 | 1.70 | 1.26 | 20 | 2.42 | 1.31 | 12 |
|  | ER_Disgust | 2.38 | 1.16 | 50 | 2.57 | 1.40 | 14 | 2.30 | 1.30 | 20 | 2.08 | 1.17 | 12 |
|  | ER_Happiness | 3.52 | 0.71 | 50 | 3.64 | 0.63 | 14 | 3.35 | 0.75 | 20 | 2.58 | 1.31 | 12 |
| Fixation Count  (FC) | FC_Anger | 55.44 | 1.42 | 50 | 59.71 | 16.08 | 14 | 54.43 | 24.93 | 18 | 35.75 | 17.72 | 13 |
|  | FC_Sandess | 57.46 | 20.49 | 50 | 58.00 | 14.17 | 14 | 52.54 | 24.77 | 18 | 21.47 | 14.74 | 13 |
|  | FC_Fear | 60.18 | 16.71 | 50 | 60.18 | 17.97 | 14 | 60.14 | 15.45 | 18 | 25.16 | 19.58 | 13 |
|  | FC_Disgust | 47.84 | 18.68 | 50 | 49.72 | 22.85 | 14 | 41.69 | 18.35 | 18 | 31.57 | 20.38 | 13 |
|  | FC_Happiness | 52.15 | 18.83 | 50 | 50.19 | 20.94 | 14 | 58.34 | 18.45 | 18 | 41.99 | 22.62 | 13 |
| Fixation Duration  (FD) (sec) | FD_Anger | 56.41 | 1.23 | 50 | 61.50 | 19.43 | 14 | 53.91 | 26.58 | 18 | 34.49 | 19.42 | 13 |
|  | FD_Sandess | 60.87 | 19.57 | 50 | 58.79 | 19.89 | 14 | 52.48 | 24.32 | 18 | 18.20 | 14.21 | 13 |
|  | FD_Fear | 63.43 | 16.82 | 50 | 60.24 | 24.59 | 14 | 61.34 | 15.76 | 18 | 25.42 | 19.13 | 13 |
|  | FD_Disgust | 48.68 | 18.36 | 50 | 52.33 | 27.11 | 14 | 42.09 | 18.51 | 18 | 32.39 | 19.61 | 13 |
|  | FD_Happiness | 53.33 | 19.60 | 50 | 50.81 | 24.98 | 14 | 59.18 | 18.49 | 18 | 41.28 | 22.58 | 13 |

**Supplementary Table 2.** Descriptive data of age, IQ, and the dependent variables (Emotion Recognition, Fixation Count, and Fixation Duration) divided by the combinations of APSD-CU cut-off and *MAOA*-uVNTR alleles. IQ= Intelligence Quotient.

|  |  | Age | | | IQ | | |
| --- | --- | --- | --- | --- | --- | --- | --- |
|  |  | **Spearman’s ρ** | ***p*-value** | **N** | **Spearman’s ρ** | ***p*-value** | **N** |
| Emotion Recognition  scores (ER) | ER_Anger | -0.020 | 0.850 | 96 | 0.039 | 0.711 | 95 |
|  | ER_Sandess | 0.054 | 0.600 | 96 | 0.053 | 0.609 | 95 |
|  | ER_Fear | -0.071 | 0.490 | 96 | 0.076 | 0.466 | 95 |
|  | ER_Disgust | 0.039 | 0.703 | 96 | 0.222 | 0.030 | 95 |
|  | ER_Happiness | -0.160 | 0.120 | 96 | -0.026 | 0.804 | 95 |
| Fixation Count (FC) | FC_Anger | 0.078 | 0.455 | 95 | 0.075 | 0.471 | 94 |
|  | FC_Sandess | 0.015 | 0.883 | 95 | 0.028 | 0.789 | 94 |
|  | FC_Fear | 0.031 | 0.764 | 95 | 0.116 | 0.266 | 94 |
|  | FC_Disgust | 0.096 | 0.353 | 95 | -0.008 | 0.938 | 94 |
|  | FC_Happiness | 0.106 | 0.307 | 95 | -0.066 | 0.525 | 94 |
| Fixation Duration  (FD) sec | FD_Anger | -0.001 | 0.992 | 95 | 0.023 | 0.824 | 94 |
|  | FD_Sandess | 0.055 | 0.599 | 95 | -0.019 | 0.852 | 94 |
|  | FD_Fear | 0.004 | 0.969 | 95 | 0.026 | 0.807 | 94 |
|  | FD_Disgust | 0.023 | 0.826 | 95 | -0.036 | 0.734 | 94 |
|  | FD_Happiness | 0.012 | 0.912 | 95 | -0.126 | 0.225 | 94 |

**Supplementary Table 3.** Search for confounding factors. Spearman’s rank correlations between the dependent variables (Emotion Recognition, Fixation Count, and Fixation Duration) and age and IQ. IQ= Intelligence Quotient.

|  |  | ADHD presence/absence | | |
| --- | --- | --- | --- | --- |
|  |  | z score | *p-*value | N |
| Emotion Recognition (ER) score | ER_Anger | -0.405 | 0.686 | 96 |
|  | ER_Sandess | -1.121 | 0.262 | 96 |
|  | ER_Fear | -0.062 | 0.951 | 96 |
|  | ER_Disgust | -1.570 | 0.116 | 96 |
|  | ER_Happiness | -1.736 | 0.083 | 96 |
| Fixation Count  (FC) sec | FC_Anger | -0.572 | 0.568 | 95 |
|  | FC_Sandess | -0.441 | 0.659 | 95 |
|  | FC_Fear | 0.024 | 0.980 | 95 |
|  | FC_Disgust | 0.580 | 0.562 | 95 |
|  | FC_Happiness | 0.551 | 0.582 | 95 |
| Fixation Duration  (FD) sec | FD_Anger | -0.053 | 0.958 | 95 |
|  | FD_Sandess | 0.045 | 0.964 | 95 |
|  | FD_Fear | 0.555 | 0.579 | 95 |
|  | FD_Disgust | 0.641 | 0.522 | 95 |
|  | FD_Happiness | 1.596 | 0.111 | 95 |

**Supplementary Table 4.** Search for confounding factors. Mann-Whitney U test between youths with and without ADHD for the dependent variables (Emotion Recognition, Fixation Count, and Fixation Duration).

|  | APSD-CU cut-off | | |
| --- | --- | --- | --- |
|  | **z score** | **p value** | **N** |
| Age | -0.890 | 0.373 | 97 |
| IQ | 0.108 | 0.914 | 96 |

**Supplementary Table 5.** Search for confounding factors. Mann-Whitney U test for age and IQ between youths with APSD-CU scores ≥ 6 and youths with APSD-CU scores < 6. IQ= Intelligence Quotient.

|  | APSD-CU < 6 | APSD-CU ≥ 6 | Odds ratio | Chi-square |
| --- | --- | --- | --- | --- |
| Absence of ADHD | 22 | 7 | 0.514 | 1.8  p= 0.180 |
| Presence of ADHD | 42 | 26 |  |  |

**Supplementary Table 6.** Search for confounding factors. Chi-square (Pearson) test to compare the frequency of ADHD diagnosis in youths with APSD-CU scores ≥ 6 with that in youths with APSD-CU scores < 6.
